# Association of Klotho gene Expression and miRNA- 339 in Schizophrenia

**DOI:** 10.1192/j.eurpsy.2023.590

**Published:** 2023-07-19

**Authors:** D. Yadav, A. Birdi, S. Tomo, N. Nebhinani, P. Sharma

**Affiliations:** ^1^Biochemistry; ^2^All India Institute of Medical Sciences, Jodhpur, India; ^3^Psychiatry, All India Institute of Medical Sciences, Jodhpur, India

## Abstract

**Introduction:**

Schizophrenia is one of the major neuropsychiatric disorders affecting 1% of the population worldwide. Neuroinflammation, neurodevelopment, and oxidative stress are some of the crucial factors that can contribute to the pathogenesis of Schizophrenia. The Klotho gene is an antiaging gene whose dysregulated expression can lead to Schizophrenia and aging-like symptoms in patients. Klotho gene expression is regulated by miRNA- 339, which might lead to expression changes of the klotho gene in schizophrenia patients.

**Objectives:**

This study aimed to determine the role of miRNA- 339-5p in the regulation of Klotho gene expression and its circulatory levels in Schizophrenia

**Methods:**

A total of 60 diagnosed patients with Schizophrenia per ICD 10 study subjects and 30 healthy controls were recruited for this study from the outpatient department of psychiatry of All India Institute of Medical Sciences, Jodhpur. Written informed consent was taken from all the study participants. All participants were given the right to withdraw from the study at any point in time. The institutional ethics committee of AIIMS, Jodhpur, approved this study. The expression analysis of the klotho gene and miRNA – 339-5p was performed using a reverse transcription polymerase chain reaction. The relative fold change expression was calculated by Livaak’s method ( 2^-double delta ct).

**Results:**

In Schizophrenia cases, serum Klotho protein levels were higher than healthy controls, though non-significant. We observed that the klotho gene was upregulated (figure 1), and miRNA- 339- 5p was downregulated (figure 2) compared to the healthy control.

**Image:**

**Figure:**
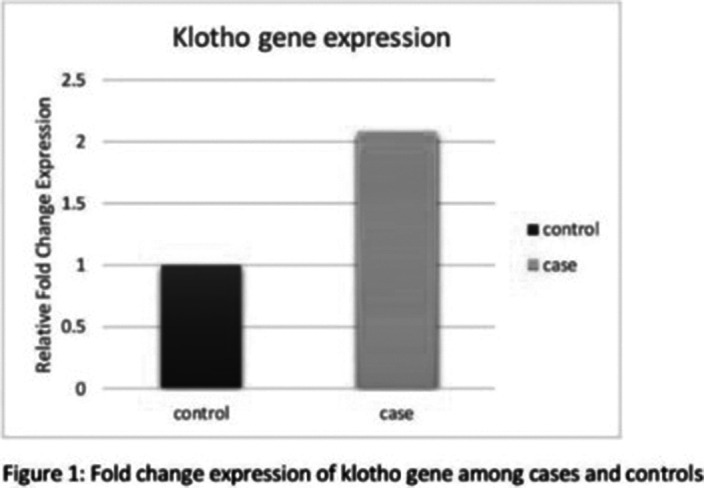

**Image 2:**

**Figure:**
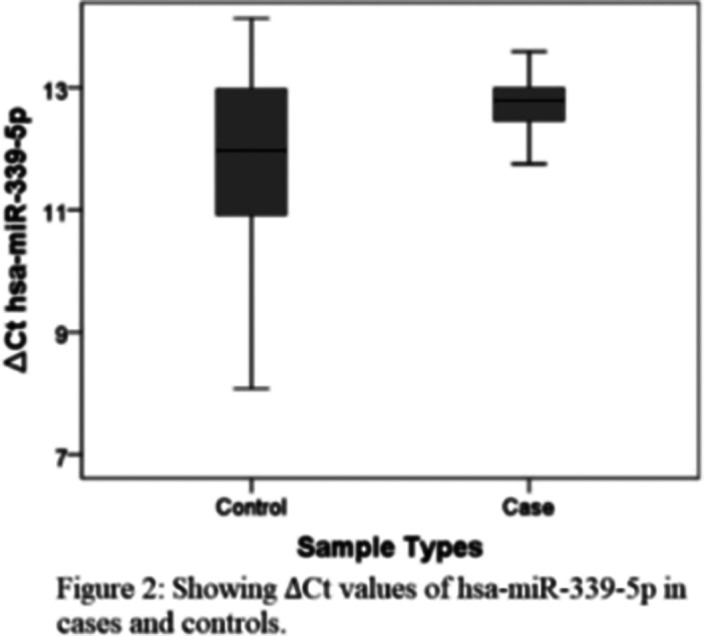

**Conclusions:**

The present study is the first to evaluate the klotho gene expression and correlate it with miRNA- 339- 5p. Our study found increased expression of the klotho gene in Schizophrenia patients as compared to the controls. In this study, we also observed that miRNA- 339- 5p was downregulated, which correlates with the klotho gene expression inversely. However, these are the preliminary findings due to less sample size and need to be replicated in the large sample size for further confirmation.

**Disclosure of Interest:**

None Declared

